# *Eocuma orbiculatum* sp. nov. (Crustacea, Cumacea, Bodotriidae) from Korean waters

**DOI:** 10.3897/zookeys.910.47143

**Published:** 2020-02-10

**Authors:** Sung-Hyun Kim, Chang-Mok Lee, Young-Hyo Kim

**Affiliations:** 1 Department of Biological Sciences, Dankook University, Cheonan 31116, South Korea Dankook University Cheonan South Korea; 2 Hanmin High School, Paju 10955, South Korea Hanmin High School Paju South Korea

**Keywords:** Bodotriidae, Cumacea, *
Eocuma
*, key, Korea, new species, taxonomy

## Abstract

A new species of Cumacea belonging to the genus *Eocuma* Marcusen was collected from the South Sea of Korea. This new species resembles *E. amakusense* Gamô, *E. hilgendorfi* Marcusen, and *E. latum* Calman in having a pair of well-developed dorso-lateral carinae on the flat carapace and similar setae pattern on the telson. The new species, however, is easily distinguished from its congeners by the pattern of dorso-lateral carina and lacking lateral horns on the carapace. The adult male of this new species is fully described. A key to the Korean *Eocuma* species is also provided.

## Introduction

The family Bodotriidae Scott, 1901 contains two subfamilies: Bodotriinae Scott, 1901 and Vaunthompsoniinae Sars, 1878 (Haye 2007). The subfamily Bodotriinae is composed of 14 genera and it is characterized by having exopods on the maxilliped 3 and pereopod 1 in both sexes, and five pairs of pleopods in males of most genera (Haye 2007; WoRMS 2019). Among the 14 genera, *Eocuma* Marcusen, 1894 is the fourth most speciose genus, after *Bodotria* Goodsir, 1843, *Cyclaspis* Sars, 1865, and *Iphinoe* Bate, 1856. To date, 31 species are known in the genus *Eocuma* (Haye 2007; WoRMS 2019), of which three have been reported from Korea: *Eocuma amakusense* Gamȏ, 1967 (Kim et al. 2017), E. cf. hilgendorfi Marcusen, 1894 (Park et al. 1998), and *E. latum* Calman, 1907 (Kim et al. 2017). In this study, we describe and illustrate a new species of *Eocuma* from Korean waters. A key to the Korean species of the genus *Eocuma* is also provided.

## Material and methods

The specimen was collected using a light-trap (Holmes and O’Connor 1988; Kim 1992) from shallow water at Hangdong Port, Wando-gun, Jeollanam-do, Korea. The collected specimen was fixed in 70–80% ethanol, moved to the laboratory, and stored in 95% ethanol. The specimen was identified with a stereomicroscope (Model SZX12; Olympus, Japan). The photographs of the whole body were taken with a microscope equipped a digital camera (eXcope T500; DIXI Science, Korea) and complemented by Helicon Focus software (Model Helicon Focus; Helicon Soft Ltd., Kharkov, Ukraine). The body length was measured from the anterior tip of the carapace to the posterior end of the pleonite 6. Lengths of the appendages were measured along the midline of each appendage. Drawing of the whole body was performed under a stereomicroscope (Model SZX12) with a drawing tube. Later, the sample was transferred to glycerin to be dissected under a stereomicroscope (Model SZX12). Drawing of the appendages were performed with a light microscope (Model BX51; Olympus, Japan). Photographs of the carapace surface were taken using a scanning electron microscope. Type specimen was deposited at the National Institute of Biological Resources (NIBR), in Incheon, Korea.

## Taxonomy

### Genus Eocuma

Taxon classificationAnimaliaCumaceaBodotriidae

Marcusen, 1894

F9DE7AFA-BA69-50C5-90BF-6446DDA4D2EA

#### Type species.

*Eocuma hilgendorfi* Marcussen, 1894.

#### Diagnosis.

Carapace may appear laterally compressed anteriorly or posteriorly in dorsal view; may be oviform posteriorly; may have dorsal median carina, dorso-lateral carinae, lateral carinae and/or lateral horns. Antenna 1 with basal article of the peduncle arcuate or straight, as long as or shorter than the other two articles combined. Maxilliped 3 basis geniculate, arcuate or straight, extended dorso-distally over ischium beyond the articulation of the ischium and merus. First pereonite visible only above lateral midline or invisible. Pereonite 2 variable in width with respect to other pereonites; may have ventro-lateral expansion overriding pereonite 3, carapace and pereonite 3 or not overriding other somites. Basis of pereopod 1 distally produced beyond insertion of ischium. Pereopod 2 without ischium; dactylus longer than propodus. Uropod peduncle much shorter than pleotelson or rami. Uropod endopod uniarticulate. Uropod exopod with proximal article shorter than distal one. Males with five pairs of pleopods.

#### Remarks.

*Eocuma* was considered similar to *Mossambicuma* Day, 1978, but could be distinguished by carapace shape and projection on the basis of the pereopod 1. Haye (2007) demonstrated, however, *Mossambicuma* to be a synonym for *Eocuma*, since *E. muradianae* Petrescu, 1998 also lacks the projection on the basis of pereopod 1 and many other species in *Eocuma* lack lateral horns. Haye (2007) supported these observations by analyzing character evolution of the family Bodotriidae, including the genus *Eocuma*. Korean species reported as *Eocuma*, including this new species, have morphological characteristics that correspond to the universal characteristics of *Eocuma* diagnosed by Haye (2007).

#### Species composition.

*Eocuma aculeatum* Day, 1978; *E. affine* Calman, 1904; *E. agrion* Zimmer, 1914; *E. amakusense* Gamô, 1967; *E. bacescui* Petrescu, 2003; *E. cadenati* Fage, 1950; *E. calmani* Fage, 1928; *E. carinocurvum* Corbera, Tirado & Martin, 2005; *E. cochlear* Le Loeuff & Intes, 1972; *E. dimorphum* Fage, 1928; *E. dollfusi* Calman, 1907; *E. elongatum* (Day, 1978); *E. ferox* (Fischer, 1872); *E. foveolatum* Day, 1978; *E. gorgasiae* Mühlenhardt-Siegel, 1996; *E. hilgendorfi* Marcusen, 1894; *E. kempi* Kurian, 1954; *E. lanatum* Le Loeuff & Intes, 1972; *E. latum* Calman, 1907; *E. longicorne* Calman, 1907; *E. muradianae* Petrescu, 1998; *E. petrescui* Patel, Haye & Kornfield, 2003; *E. rosae* Corbera & Galil, 2007; *E. sarsii* (Kossmann, 1880); *E. spiniferum* Gamo, 1976; *E. stelliferum* Calman, 1907; *E. striatum* Kurian & Radha Devi, 1983; *E. taprobanicum* Calman, 1904; *E. travancoricum* Kurian, 1951; *E. victoriae* (Mühlenhardt-Siegel, 2003); and *E. winri* Day, 1978.

### Eocuma orbiculatum sp. nov.

Taxon classificationAnimaliaCumaceaBodotriidae

1628A1D8-8728-5B37-B36E-13F6F601D87C

http://zoobank.org/478C96E3-96B6-4E3F-90DE-81874690FF3B

**Figure 1.**
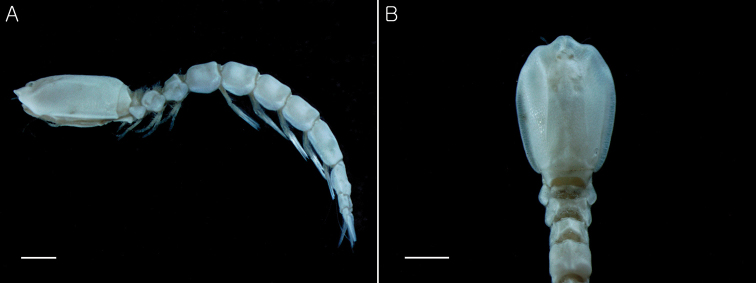
*Eocuma orbiculatum* sp. nov., holotype, adult male, 11.92 mm. **A** Lateral view **B** cephalothorax, dorsal view. Scale bars: 1.0 mm (**A, B**).

**Figure 2.**
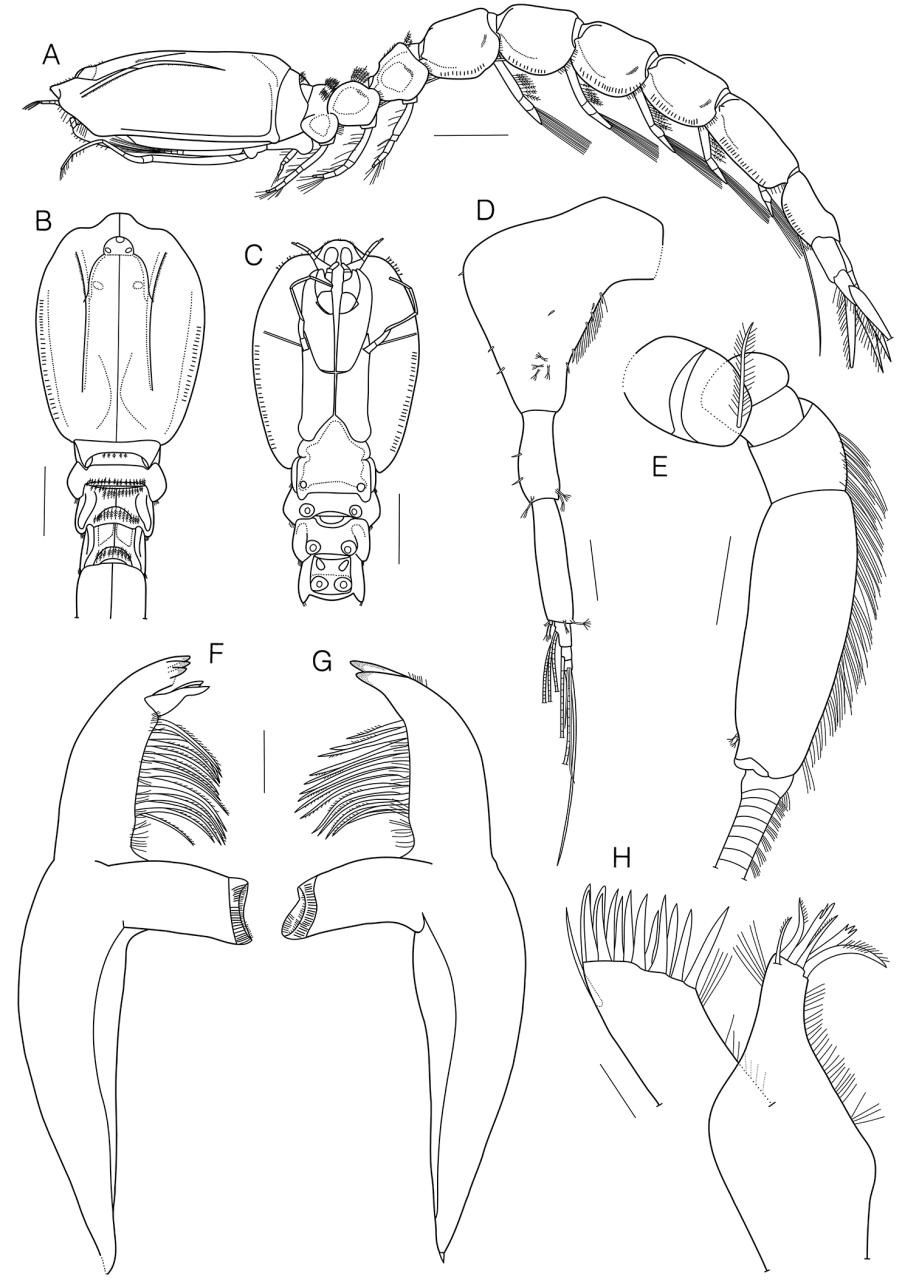
*Eocuma orbiculatum* sp. nov., holotype, adult male, 11.92 mm. **A** Habitus, lateral view **B** cephalothorax, dorsal view **C** cephalothorax, ventral view **D** antenna 1 **E** antenna 2 **F** left mandible **G** right mandible **H** maxilla 1. Scale bars: 1.0 mm (**A–C**), 0.2 mm (**D**), 0.1 mm (**C, E, F**), 0.05 mm (**G**).

**Figure 3.**
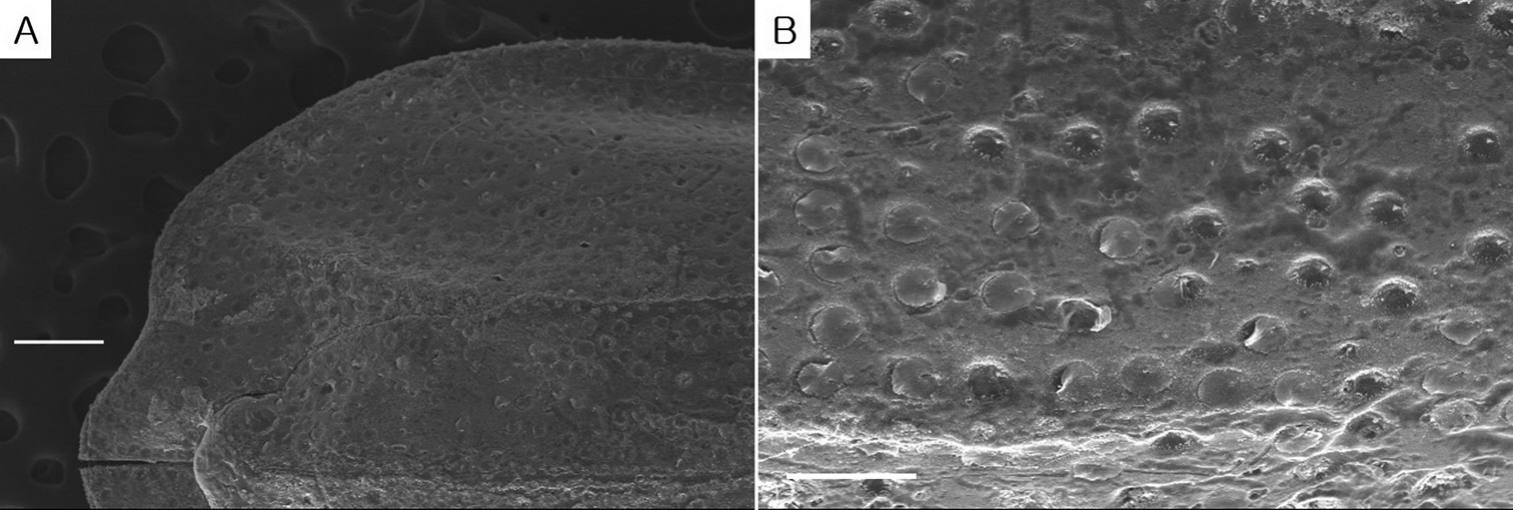
*Eocuma orbiculatum* sp. nov., holotype, adult male, 11.92 mm. **A, B** Carapace, dorsal view. Scale bar: 250 μm (**A**), 125 μm (**B**).

**Figure 4.**
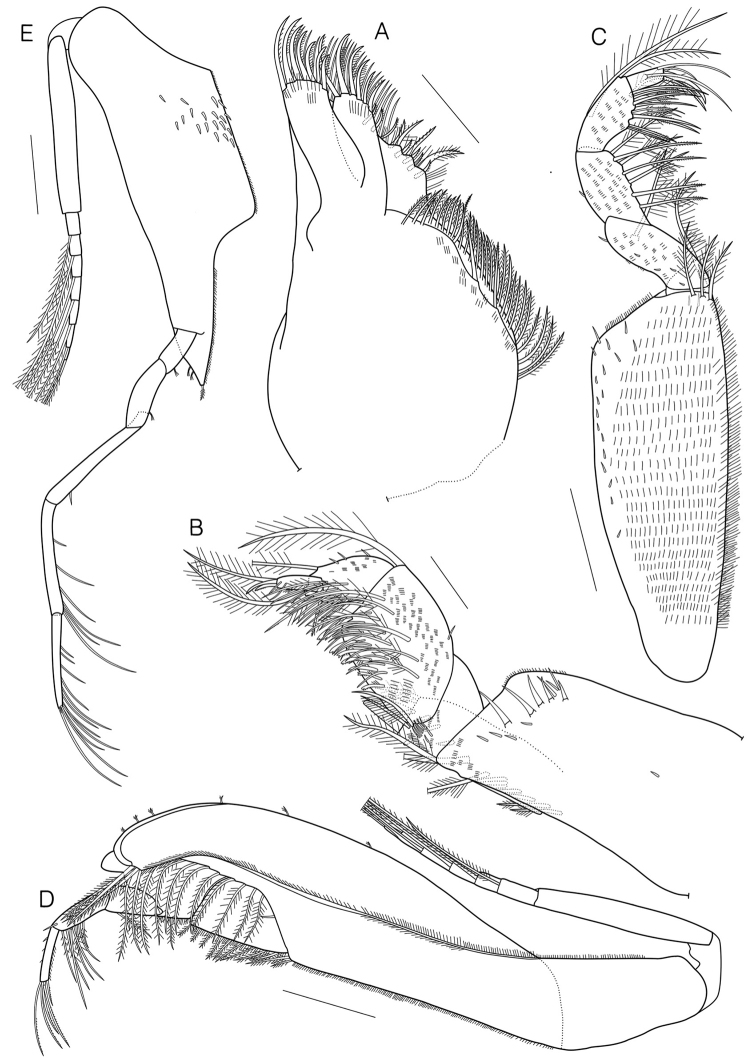
*Eocuma orbiculatum* sp. nov., holotype, adult male, 11.92 mm. **A** Maxilla 2 **B** maxilliped 1 **C** maxilliped 2 **D** maxilliped 3 **E** pereopod 1. Scale bars: 0.3 mm (**D, E**), 0.2 mm (**C**), 0.1 mm (**A, B**).

**Figure 5.**
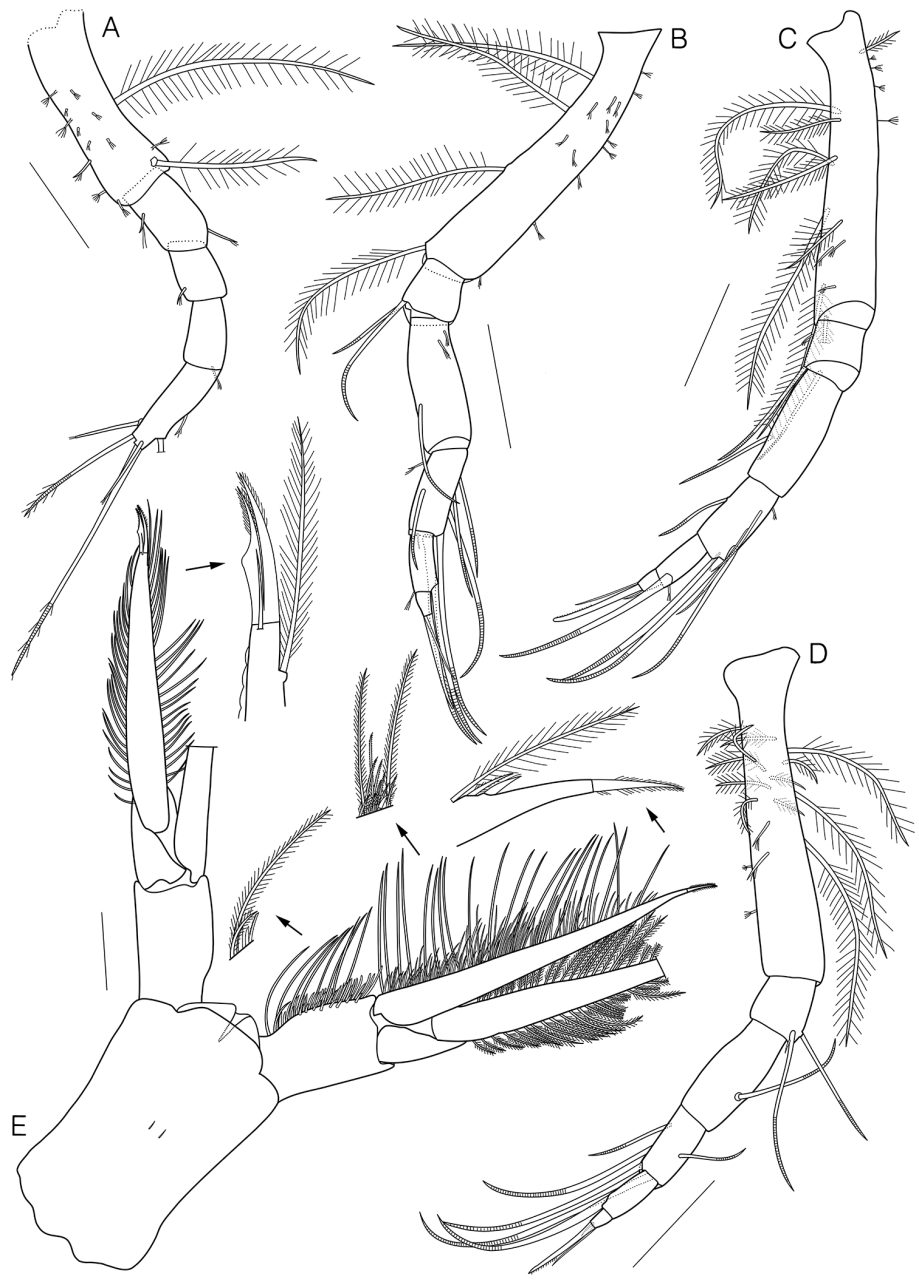
*Eocuma orbiculatum* sp. nov., holotype, adult male, 11.92 mm. **A** Pereopod 2 **B** pereopod 3 **C** pereopod 4 **D** pereopod 5 **E** pleotelson. Scale bars: 0.3 mm (**E**), 0.2 mm (**B–D**), 0.1 mm (**A**).

Figures 1 , 2 , 3 , 4 , 5

#### Type material.

Holotype: adult male, 11.92 mm, NIBRIV0000812748, Hangdong Port, Sedong-ri, Wando-gun, Jeollanam-do, Korea, 34°23'38.2"N, 126°50'29.3"E, Y.H. Kim, 22 June 2008.

#### Description.

Holotype, adult male, NIBRIV0000812748.

Length: 11.92 mm, excluding uropods. Carapace (Figs 1A, B, 2A–C, 3A, B) 0.26 × body length, 1.4 × width; surface covered with minute shallow pits; lateral margins of carapace, pereon, and pleon lamellate; pseudorostrum a little in advance of antero-lateral horns with rounded apices, without lateral horns; dorsal median carina extending from eye-lobe to posterior margin of carapace; a pair of dorso-lateral carinae well-marked, extending from near the antero-lateral horns to 4/5 way of carapace; ocular lobe (Fig. 2B) with 3 lenses. Pereon (Fig. 2A, B) 0.7 × carapace. Pleon (Fig. 2A) 1.3 × cephalothorax.

Antenna 1 (Fig. 2D) peduncle 3-articulate; article 1 flattened, broader at base, with several hair-like, 10 short simple, and 4 complex pedunculate setae; article 2 0.4 × article 1, with 2 short simple and 3 complex pedunculate setae; article 3 1.4 × article 2, with 2 complex pedunculate setae distally. Main flagellum 3-articulate; article 1 with 3 aesthetascs; article 2 with 1 aesthetasc; article 3 with 2 simple setae, 1 long simple seta, and 1 aesthetasc terminally. Accessory flagellum minute, with 1 short simple and 2 complex pedunculate setae.

Antenna 2 (Fig. 2A, E) extending beyond pleotelson; peduncle 5-articulated; article 2 with 1 or 2 plumose setae; articles 4–5 with numerous simple setae, article 5 with 2 complex pedunculate setae dorso-distally.

Left mandible (Fig. 2F) with row of several hair-like and 14 setae; incisor with 4 teeth; *lacinia mobilis* with 3 teeth. Right mandible (Fig. 2G) with row of several hair-like and 14 setae; incisor with 2 teeth.

Maxilla 1 (Fig. 2H) outer endite with a few hair-like setae medially, 1 simple seta laterodistally, 13 stout simple setae terminally; inner endite with several hair-like setae medially, a few hair-like setae laterodistally, 4 microserrate and 2 stout tricuspid setae terminally; palp broken, with 2 setae.

Maxilla 2 (Fig. 4A) broad endite with several hair-like, 26 plumose, and 3 microserrate setae medially, 6 plumose, 25 simple, 11 microserrate, 2 plumo-microserrate, and 1 pappo-serrate setae terminally; outer endite with a few hair-like and 7 stout microserrate setae terminally; inner endite with 6 stout microserrate setae terminally.

Maxilliped 1 (Fig. 4B) basis with a few hair-like setae mediodistally, 1 long plumose seta on medial distal corner, 13 simple setae on lateral surface, a few hair-like setae laterodistally; medial lobe with 1 coupling hook and 8 plumo-microserrate setae medially, 3 stout simple setae on medial distal surface, 1 plumose and 2 stout simple setae terminally; ischium absent; merus with a few hair-like, 8 short simple, and 2 long plumose setae; carpus 2.1 × merus, with 21 plumose and 8 stout setae medially, numerous hair-like setae on lateral surface, 1 short simple seta laterally, 1 long plumose seta on laterodistal corner; propodus 0.5 × carpus, with a few hair-like, 20 plumose, 1 simple, 1 microserrate, and 1 plumo-microserrate setae medially, a few hair-like and 3 short simple setae medially, 3 long plumose setae distally; dactylus 0.7 × propodus, a few hair-like and 2 simple setae medially, 2 stout microserrate setae terminally.

Maxilliped 2 (Fig. 4C) basis 1.4 × remaining articles combined, with numerous hair-like setae medially, 2 short simple and 4 long plumose setae mediodistally, numerous hair-like setae on lateral surface, 18 short simple and several hair-like setae laterally; ischium short, unarmed; merus 0.3 × basis, with 1 long plumose seta on medial distal surface, several hair-like and 4 short simple setae on lateral surface, a few hair-like and 1 short simple setae laterally; carpus 0.7 × merus, with 3 plumose and 4 plumo-microserrate setae medially, several hair-like setae on lateral surface, 1 short simple seta laterally; propodus 0.8 × carpus, with 2 microserrate, 10 plumo-microserrate, and 1 plumose setae medially, several hair-like setae on lateral surface, 2 long plumose setae laterally; dactylus 0.5 × propodus, with 1 long simple seta on lateral surface, 2 simple, 1 microserrate, and 1 stout microserrate setae terminally.

Maxilliped 3 (Fig. 4D) basis 1.4 × remaining articles combined, with numerous hair-like setae medially, 2 plumose setae on medial distal corner, numerous hair-like setae on medial surface and lateral margin, 1 short simple seta with subterminal setules laterally; produced distally to border between merus and carpus, with 1 short simple and 14 plumose setae medially, 5 short simple setae with subterminal setules laterally; ischium 0.2 × basis, with several hair-like and 9 plumose setae medially; merus subequal to ischium, with several hair-like and 1 plumose setae medially; carpus 0.5 × merus, with 1 plumose seta medially; propodus 1.1 × carpus, with 1 short simple and 7 plumose setae medially, 1 short simple seta laterodistally; dactylus 0.8 × propodus, with a few hair-like setae medially, 1 short simple and 2 hair-like setae laterally, 5 microserrate setae terminally; exopod shorter than basis.

Pereopod 1 (Fig. 4E) basis 0.9 × remaining articles combined, with numerous hair-like setae medially, 19 short simple setae on lateral surface, 2 complex pedunculate setae laterodistally, 1 short plumose seta on terminal point; ischium 0.1 × basis, with 1 complex pedunculate seta mediodistally; merus 1.7 × ischium, with 1 complex pedunculate seta mediodistally; carpus 1.6 × merus, with 1 short simple seta mediodistally; propodus subequal to carpus, with 6 simple setae medially; dactylus 0.9 × propodus, with 9 simple setae medially, 1 short simple and 2 simple setae terminally; exopod shorter than basis.

Pereopod 2 (Fig. 5A) length 0.3 × basis of pereopod 1, basis fused with ischium, 0.6 × remaining articles combined, with 2 long plumose and 1 complex pedunculate setae medially, 3 complex pedunculate setae on lateral surface, 7 complex pedunculate setae and 1 short simple seta with subterminal setules laterally; merus 0.4 × basis, with 1 short simple seta with subterminal setules mediodistally, 1 short simple seta with subterminal setules lateroproximally; carpus 0.6 × merus, with 1 short simple seta with subterminal setules laterodistally; propodus 1.4 × carpus, with 1 short simple seta with subterminal setules mediodistally; dactylus 1.5 × propodus, with 1 short simple seta with subterminal setules and 1 broken seta medially, 1 simple seta with terminal setules laterally, 1 plumo-annulate seta, 1 long plumo-annulate seta with single subterminal setule, and 1 simple seta with terminal setules terminally.

Pereopod 3 (Fig. 5B) basis 0.9 × remaining articles combined, with 5 complex pedunculate setae and 2 short simple setae with subterminal setules medially, 6 short simple setae with subterminal setules on lateral surface, 4 long plumose setae laterally; ischium 0.2 × basis, with 2 annulate setae laterodistally; merus 2.7 × ischium, with 2 short simple setae with subterminal setules medioproximally, 1 annulate seta laterally; carpus 0.7 × merus, with 3 annulate setae medially, 1 short simple seta with subterminal setules and 1 annulate seta laterally, 1 short simple and 2 long annulate setae on medial distal margin; propodus 0.7 × carpus, with 1 long annulate seta on medial distal corner, 1 complex pedunculate seta laterodistally; dactylus 0.4 × propodus, with 1 simple and 1 stout microserrate setae terminally.

Pereopod 4 (Fig. 5C) basis 0.9 × remaining articles combined, with 1 plumose and 4 complex pedunculate setae medially, 8 plumose setae and 3 short simple setae with subterminal setules laterally; ischium 0.2 × basis, with 1 short simple seta with subterminal setules laterally, 2 annulate setae on distal margin; merus 2.3 × ischium, with 1 annulate seta laterodistally; carpus 0.7 × merus, with 3 long annulate setae and 1 short simple seta with subterminal setules medially, 1 short simple and 2 long annulate setae on medial distal corner, 1 annulate seta laterally; propodus 0.7 × carpus, with 1 long annulate and 1 complex pedunculate setae mediodistally; dactylus 0.4 × propodus, with 2 simple and 1 stout microserrate setae terminally.

Pereopod 5 (Fig. 5D) basis subequal to remaining articles combined, with 3 long plumose, 9 plumose, 2 complex pedunculate setae, and 2 short simple setae with subterminal setules; ischium 0.2 × basis, with 1 short simple and 2 annulate setae laterodistally; merus 2.0 × ischium, with 1 annulate seta laterally; carpus 0.6 × merus, with 4 annulate setae medially, 1 annulate seta laterally; propodus 0.8 × carpus, with 1 long annulate seta on medial distal corner; dactylus 0.3 × propodus, with 2 simple and 1 stout microserrate setae terminally.

Uropod (Fig. 5E) peduncle 0.4 × pleotelson, with 9–10 plumose, 14–16 stout short simple, and 35–40 microserrate setae medially. Endopod uniarticulate, 3.2 × peduncle, with 21 plumose, 77 plumo-microserrate, and 3 stout short simple setae medially, tip with setulate seta. Exopod biarticulated, subequal to endopod, article 1 unarmed; article 2 with 23 plumose setae medially, 21 plumose setae laterally, 1 simple seta distally, tip with 2 curved setulate setae.

#### Remarks.

This new species resembles *Eocuma amakusense* Gamô, 1967, *E. hilgendorfi* Marcusen, 1894, and *E. latum* Calman, 1907 in having a pair of well-developed dorso-lateral carinae on the flat carapace and similar setae pattern on the telson. *Eocuma orbiculatum* sp. nov., however, is easily distinguished from its congeners by the pattern of dorso-lateral carina and lacking lateral horns on the carapace. This difference also applies when considering growth, geographical distribution and individual variations (Table 1). Comparison between the species was done assuming that *E. orbiculatum* sp. n and E. cf. hilgendorfi (by Park et al. 1998) subadult male was the same species, since information of *E. hilgendorfi* adult male was not suggested in previous studies. *Eocuma orbiculatum* sp. n is distinguished from the specimen of Park et al. (1998) by the combination of the following features (E. cf. hilgendorfi condition in parentheses): 1) a pair of dorso-lateral carinae from near the near apices of the antero-lateral horns extending to approximately 0.8 of the carapace length (vs. from ocular lobe to the posterior margin of carapace); 2) carapace without lateral horn (vs. with lateral horns); 3) basis of pereopod 1 with 19 short simple setae on ventral surface (vs. without short simple seta); 4) carpus of pereopod 1.6 × merus length (vs. 2.3 × merus length); 5) medial margin of uropod peduncle and endopod with plumose, stout short simple, and microserrate setae (vs. with plumose setae on endopod, with plumose setae and 3 spaced teeth on exopod); 6) uropod exopod with plumose setae laterally (vs. without seta). Even considering growth and individual variations, many differences were found in carapace shape, carpus length, and setal pattern of the telson. In addition, E. cf. hilgendorfi (by Park et al. 1998) is considered to be a different species, as it shows many morphological differences from *E. hilgendorfi* reported by Zimmer (1903). Additional samples shall be obtained and identified.

**Table 1.** Comparison of morphological characteristics among *Eocuma orbiculatum* sp. nov. and related species (based on males).

Characteristics and distribution	*orbiculatum* (adult)	*amakusense* (adult)	*amakusense* (adult)	*hilgendorfi* (subadult)	*cf. hilgendorfi* (subadult)	*latum* (adult)	*latum* (adult)	*latum* (adult)	*latum* (adult)	* latum *	*latum* (subadult)
Body length	11.92 mm	11.9 mm	11.4 mm	10 mm	11.4 mm	8.5 mm	6 mm	12.8–13.5 mm	13 mm	unknown	10.7 mm
Carapace, antero-lateral horns	with round apices	with round apices	with round apices	with pointed apices	with round apices	with round apices	with round apices	with round apices	with round apices	with round apices	with round apices
dorso-lateral carinae	extending from near the antero-lateral horns to 4/5 way of carapace	extending from near the antero-lateral horns to posterior margin	extending from near the antero-lateral horns to near posterior margin	extending from ocular lobe to posterior margin	extending from ocular lobe to posterior margin	extending from ocular lobe to posterior margin	extending from ocular lobe to posterior margin	extending from ocular lobe to posterior margin	extending from near the antero-lateral horns to near posterior margin	extending from ocular lobe to posterior margin	extending from ocular lobe to posterior margin
lateral horns	without lateral horn	with lateral horn, convex lateral sides of the carapace	with lateral horns, rounded	with lateral horns, very vertically deep	with lateral horns	with forwarded lateral horns	with forwarded lateral horns	with forwarded lateral horns	with forwarded lateral horns	with forwarded lateral horns	with forwarded lateral horns
Eye-lobe, lens	with 3 lenses	with 5 lenses	with 5 lenses	with 3 lenses	with 3 lenses	with 8 lenses	with 3 lenses	with 3 lenses	with 3 lenses	unknown	with 3 lenses
Pereopod 1, basis ventral surface	with 19 short simple setae	with 27 short simple setae	with 10 short simple setae	unknown	without short simple seta	with 10 short simple setae	without short simple seta	with short simple setae	unknown	without short simple seta	with blunt simple setae
carpus length	1.6 × merus	2.6 × merus	2.7 × merus	unknown	2.3 × merus	2.3 × merus	2.4 × merus	1.7 × merus	unknown	2.4 × merus	1.4 × merus
dactylus length	0.9 × propodus	0.5 × propodus	0.6 × propodus	unknown	0.9 × propodus	0.5 × propodus	0.6 × propodus	0.7 × propodus	unknown	0.7 × propodus	subequal to propodus
Pereopod 2 merus, mediodistally	without protuberance	with protuberance	with protuberance	unknown	without protuberance	unknown	with protuberance	unknown	unknown	unknown	without protuberance
Uropod peduncle, medial margin	with plumose, stout short simple, and microserrate setae	with hairy and setose	with plumose, small simple, and microserrate setae	with plumose and hair-like setae	with plumose hairs	with plumose setae and short spines	with plumose, short simple, and other setae	with plumose and other setae	unknown	with plumose setae	with plumose setae
Uropod endopod, medial margin	with plumose, plumo-microserrate, and stout short simple setae	with hairy, setose, and teeth	with plumose, plumo-microserrate, and small simple setae	with plumose and hair-like setae	with plumose hairs and 3 spaced teeth	with plumose setae and spines	with plumose, short simple, and other setae	with plumose, short simple, and other setae	unknown	with plumose and short simple setae	with plumose and short simple setae
Uropod exopod, lateral margin	with plumose setae	with plumose hairs	with plumose setae	without seta	without seta	without seta	serrated, with plumose seta	with plumose setae	unknown	without seta	without seta
Distribution	South Sea (present study)	Tomoe Bay (Gamô 1967)	South Sea (Kim et al. 2017)	Enoshima (Zimmer 1903)	Yellow Sea (Park et al. 1998)	Annam (Fage 1945)	Indo-Chinese Sea (Zimmer 1952)	Sagami Bay (Gamô 1958)	Sagami Bay (Gamô 1963)	Jiaozhou bay, Yellow Sea (Liu and Liu 1990)	South Sea (Kim et al. 2017)

#### Etymology.

The new species name *orbiculatum* is a Latin word, meaning ‘round’, alluding to the absence of a horn on the lateral margin of the carapace and the round appearance of the carapace in dorsal view.

#### Distribution.

The new species was collected in Hangdong Port, Sedong-ri, Wando-gun, Jeollanam-do, Korea, muddy bottom, 2–5 m depth.

##### Key to the Korean *Eocuma* species

**Table:** 

1	Carapace lacking lateral horn; a pair of dorso-lateral carinae extending to approximately 0.8 of the carapace length	***E. orbiculatum* sp. nov.**
–	Carapace with lateral horn; a pair of dorso-lateral carinae extending to the posterior margin of carapace	**2**
2	Carapace, lateral horn obtuse and rounded	***E. amakusense***
–	Carapace, lateral horn prominent and acute	**3**
3	Pereopod 1, basis length much longer than remaining articles combined and without short simple setae on lateral surface	**E. cf. hilgendorfi**
–	Pereopod 1, basis length equal to remaining articles combined and with several short simple setae on lateral surface	***E. latum***

## Supplementary Material

XML Treatment for Eocuma

XML Treatment for Eocuma orbiculatum
